# On the Application of the Homœopathic Materia Medica

**Published:** 1889-02

**Authors:** 


### ON THE APPLICATION OF THE HOMŒOPATHIC MATERIA MEDICA.

May I ask Dr. Hitchcock what he means when he says, in the November number of THE HOMŒOPATHIC PHYSICIAN, page 587, "I find it the best way not to attempt to memorize, but to absorb," what? I do not believe the worthy Doctor when he asserts he could not give the characteristics of a dozen remedies, as he certainly fairly earned his homœopathic diploma by passing a thorough examination of materia medica and clinical medicine. Materia medica is one of the four branches (materia medica, clinical medicine, surgery, and obstetrics) which has to be passed with seventy-five per cent. to earn a diploma, and some students failed to pass on account of their deficiency in materia medica. We all know it is an impossibility to memorize our materia medica, and if one had such a prodigious memory, it would be of very little use to him. This branch of medical art and science must be studied in such a manner that the student or practitioner grasps the pivotal spirit of the drug in order to make a remedy out of it; we must find out the peculiarity of such drug, its uncommon, characteristic symptoms, by which it differs from every other drug, and through which it becomes an individuality, an entity, and all the other common symptoms will naturally find their place around that key-note. This is the science of materia medica, and to be enabled to grasp this spirit in our drugs, we must be well versed in all other branches of our profession. If Dr. Hitchcock calls this absorption, well and good, or otherwise I

would beg to be shown the way for a better study of our materia medica.

The art of prescribing is the most difficult part, and Hahnemann teaches justly that we must be fully convinced of our selection, hence the use of a repertory at the bedside cannot be too highly recommended ; but when one has to make from twenty-five to thirty visits in the city, or where the country practitioner rides daily a circuit of many miles, he has hardly time enough to study up every case thoroughly at the bedside, nor has he space enough in his buggy to carry a little library and a small drug-store from the $\theta$ to the MM. Let us be candid, and do not mislead or frighten the young beginner. Chronic cases can be fully studied out at the midnight lamp, and Saccharum lactis will not spoil a case. In acute cases, even the most strict Hahnemannian sees his way from the very start by taking up the corresponding symptoms of the case and of the drug. Seasons, epidemic and epidemic peculiarities have to be taken into consideration, it is often wonderful how one remedy at a peculiar time covers so many different cases. Grauvogl's constitutions are no idle dreams, and this peculiar, uncommon state of the patient has to be taken into consideration, though our repertories fail to give the remedy suitable to their use. In fact, our repertories are still more than imperfect, and for the same symptoms they differ in not naming the same remedies. Perhaps salvation will come from Kansas City.

Alas! we must, also, individualize our patients. A lady of one of the wealthiest families in the State and suffering from a host of chronic ailments, was brought to me for treatment. Every symptom was carefully noted down in my case-book, and I promised to study out her case to the best of my ability. I never saw the case again, for my good lady told her friends, if that old man does not know enough to prescribe, she had no use for him—and I made a note of it in my cranium, and Saccharum lactis rose still higher in my estimation.

The key-note system has been misused and abused, and too often the totality of the symptoms on that very account neglected. What one considers the prominent, peculiar symptom of a case, may be, after all, an error of judgment, and prescribing for this prominent peculiar symptom, though studied out at the bedside, may lead one astray. I never felt more pleased in my life, when I heard a Hering acknowledge his failures ; and when he with his stupendous memory made mistakes, may we not in humility acknowledge: *in omnibus caritas* and may the good Lord forgive our shortcomings.

